# Supplementary material for: Drosophila MESR4 Gene Ensures Germline Stem Cell Differentiation by Promoting the Transcription of bag of marbles
Source: Cells. 2022 Jun 28;11(13):2056. doi: 10.3390/cells11132056 (PMC9265997; doi:10.3390/cells11132056)
Supplement: Supplementary file 1 [file cells-11-02056-s001.zip › cells-1772068-supplementary.pdf]

Supplementary Materials

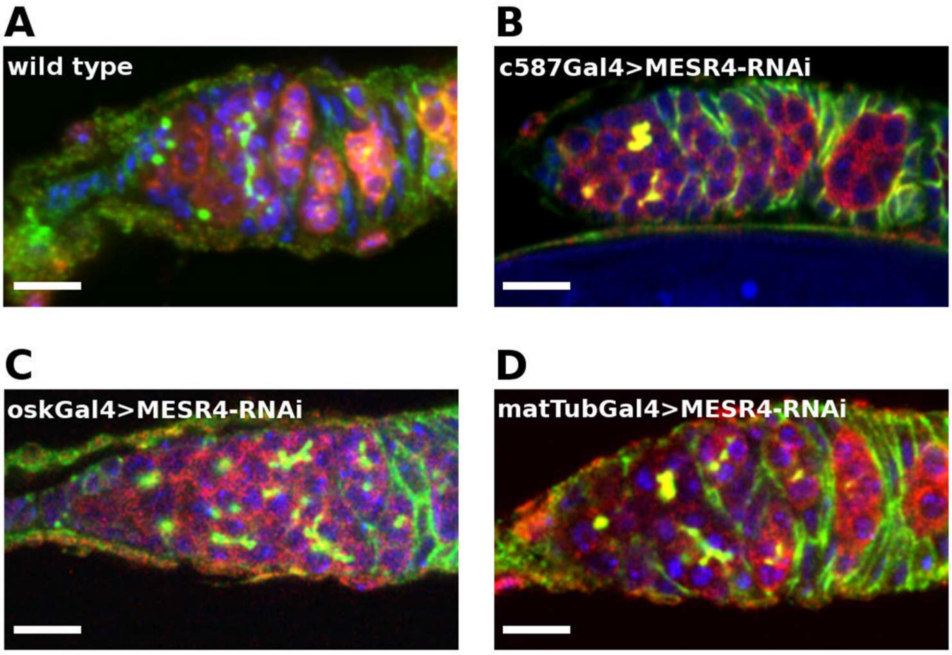

Figure S1. ***MESR4*** **function is required for the early germ cell development exclusively in the niche**.

(A-D) Immunostaining of wild type (A,), c587Gal4>*MESR4*-RNAi (B), oskGal4>*MESR4*-RNAi (C), and matTubGal4>*MESR4*-RNAi niches showing normal germ cell differentiation. Spectrosomes and fusomes are labelled with HTS (green), germ cells are labelled for Vasa (red); DAPI is blue. Scale bars are 10 µm.

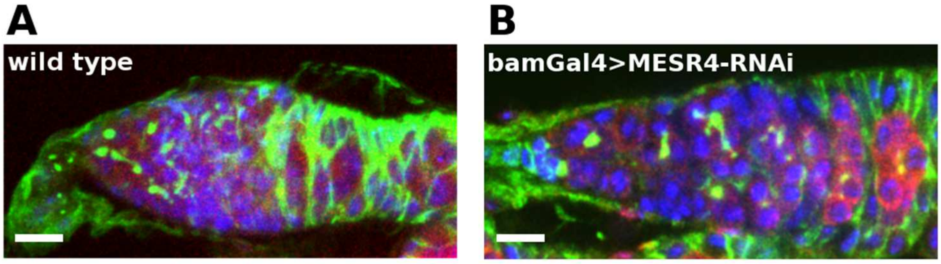

Figure S2. ***MESR4*** **function is not required in** ***bam*** **expressing CBs.**

(A,B) Immunostaining of wild type (A) and bamGal4>*MESR4*-RNAi (B) niches showing normal germ cell differentiation. Spectrosomes and fusomes are labelled with HTS (green), germ cells are labelled for Vasa (red); DAPI is blue. Scale bars are 10 µm.
